# Estimating the Benefit of Transplant Over Dialysis in Candidates Over 55 Years

**DOI:** 10.34067/KID.0000000710

**Published:** 2025-01-22

**Authors:** Margaret R. Stedman, Patrick C. Ahearn, Christine K. Liu, Glenn M. Chertow, Jane C. Tan

**Affiliations:** 1 Division of Nephrology, Department of Medicine, Stanford University School of Medicine, Stanford, California; 2 Division of Primary Care and Population Health, Department of Medicine, Stanford University School of Medicine, Stanford, California; 3 Geriatric Research and Education Clinical Center, Veteran Affairs Palo Alto Health Care System, Palo Alto, California; 4 Department of Epidemiology and Population Health, Stanford University School of Medicine, Stanford, California

**Keywords:** dialysis, epidemiology and outcomes, kidney transplantation, mortality risk, survival, transplant outcomes

## Abstract

**Key Points:**

- Older kidney transplant candidates experience a gain in life years from transplantation.
- More updated models using a contemporary cohort are needed to guide current clinical practice for older adults.
- The magnitude of gain in life years from transplant has diminished in recent years, likely driven by candidate characteristics and policy changes.

**Background:**

Life Years from Transplant (LYFT) is a measure of the predicted difference between the expected lifespan with and without a kidney transplant. The metric was originally proposed in 1999; since then, demographics of the kidney transplant candidate population have materially changed.

**Methods:**

Using contemporary Scientific Registry of Transplant Recipients data, we propose more sophisticated methods for estimating LYFT with a focus on older kidney transplant candidates, a growing sector of the current candidate pool. We examine trends in predicted LYFT from 1995 to 2020.

**Results:**

We show that among older patients on the deceased donor waitlist, transplant remains a better option compared with dialysis (overall LYFT=5 years). LYFT trends have diminished modestly (by <1 life year) over time, in part related to efforts to enhance access to transplantation through intercurrent policy changes.

**Conclusions:**

Updated LYFT estimates remain informative clinical measures that can support patient-centered decision-making. However, less homogenous metrics with meaningful disaggregation are needed to inform institutional evaluation and policy change. Models should be repeatedly evaluated as demographics of the candidate pool evolve.

## Visual Abstract

**Figure:**
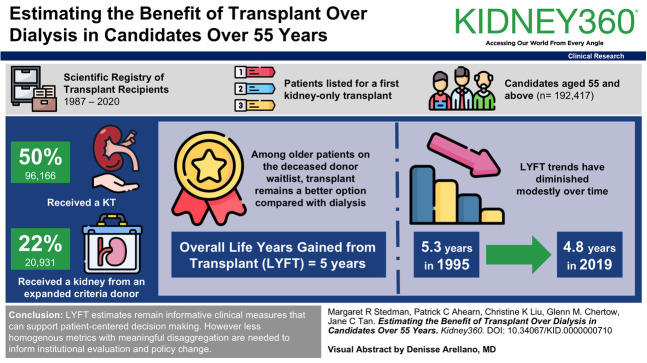

## Introduction

The Life Years from Transplant (LYFT) metric is the difference in expected survival with kidney transplant versus the expected survival without the transplant.^1^ LYFT is used in two key ways: (*1*) to formulate health policies for kidney transplantation, *e.g*., the 2014 kidney allocation system for allocation for deceased donors^2,3^, and (*2*) to communicate risks and benefits of kidney transplantation to the public, including transplant candidates and their families.^4^

The utility of LYFT hinges on accurate estimation of the expected lifespan. Originally published by Wolfe *et al.* in 1999, LYFT incorporated the characteristics of kidney transplant candidates of that era.^1,5,6^ In 1999, 33% of kidney transplant candidates were 55 years or older; in 2021, the proportion was 55%.^7^ Moreover, kidney transplant candidates older than 55 years are more willing than younger patients to accept a high kidney donor profile index (KDPI) deceased donor transplant, which may expedite transplantation. According to the Scientific Registry of Transplant Recipients (SRTR), 73% of candidates willing to accept kidneys with KDPI >85 were aged 55 and older. Changes in the demographics of kidney transplant candidate age and higher reliance on high KDPI kidneys have likely altered the LYFT metric over the past quarter-century.^8–10^ In this study, we aimed to recalculate LYFT using contemporary data and updated statistical approaches. We hypothesized that the LYFT would be greater now than when initially evaluated.

## Methods

### Study Cohort

This study used data from the Standard Analysis Files of the SRTR (1987–2020, accessed August 12, 2022). The SRTR data system includes data on all donor, waitlisted candidates, and transplant recipients in the United States, submitted by the members of the Organ Procurement and Transplantation Network (OPTN). The Health Resources and Services Administration (HRSA), an agency of the US Department of Health and Human Services, provides oversight to the activities of the OPTN and SRTR contractors. We identified candidates aged 55 and older who were listed for a first kidney-only transplant using the SRTR data (*n*=337,754). We next excluded preemptive transplant recipients and/or others with missing basic demographic information. Following the method of Wolfe *et al.*, we created two groups of waitlisted patients: those remaining on dialysis and those who underwent kidney transplant.^2^ Transplant recipients contributed time to both dialysis and transplant comparison groups. All transplant candidates were followed from the listing date until date of death. Transplant recipients contributed additional time to the dialysis group from the listing date until the date of transplant. Hence, patients who remained on dialysis contributed their entire time to the dialysis group, while patients who received a transplant contributed their entire time to the transplant group and a subset of their time to the dialysis group. We censored all patients on March 1, 2020, corresponding to the widespread recognition of the coronavirus disease 2019 pandemic, which abruptly changed the dynamics of kidney transplantation. We obtained death dates from the SRTR; persons 90 years and older were presumed deceased.

### Covariates

We stratified models by 5-year age increments (55–60, 61–65, 66–70, and older than 71 years), transplant recipients by expanded criteria donor (ECD) kidney status (standard, ECD, or no transplant), and KDPI (<20%, 20%–85%, >85%). We adjusted the main variable of interest (transplant status) for candidate designated race and ethnicity (non-Hispanic White, Non-Hispanic Black, Hispanic, Asian, and other), sex, cause of kidney failure (diabetes, hypertension, GN, and other), Quételet (body mass) index (body mass index [BMI]), waitlist year, panel-reactive antibody (PRA), serum albumin, dialysis vintage (time between the start of dialysis and waitlist), selected comorbid conditions (diabetes, peripheral arterial disease, malignancy, chronic obstructive pulmonary disease, and cerebral vascular disease), and the dates of important policy changes (2002 for ECD and 2015 for KDPI). BMI was categorized as per the World Health Organization as <18.5, 18.5 to <25, 25 to <30, 30 to <35, and >35 kg/m^2^.^11^ Owing to potential confusion in documenting weight (whether in kilograms or pounds), we imputed new values for BMIs in excess of 50 kg/m^2^. Waitlist year was modeled flexibly with cubic splines. PRA was the maximum value found in the PRA history and candidate files and was categorized into 0% to <1%, 1%–20%, >20%–80%, and >80%. Serum albumin (g/dl) was retained as a continuous variable in the model. Missing covariate information was imputed using multiple imputation by chained equations (MICE) specifying five datasets and a Nelson Aalen estimator for the survival function. We did not impute missing data for ECD and KDPI.

### Specific Cases

We demonstrate the utility of the model by projecting LYFT for four example cases: a non-Hispanic White man with PRA 0%–1%, a non-Hispanic Black woman with PRA 1%–20%, a non-Hispanic Black woman with PRA >80%, and a Hispanic man with PRA 20%–80%. All cases had a BMI of 30–35 kg/m^2^, 1-year history of dialysis at the time of listing, and serum albumin 3.5–4.0. All simulated patients had a history of diabetes except the Hispanic man. We examined the trend in LYFT in each of these cases for listing years between 1995 and 2020. We stratified trends in LYFT by age, KDPI, and ECD status of the transplant compared with no transplant. We calculated kidney donor risk index using formulas provided by “A guide to calculating and interpreting the KDPI”^12^ and mapped kidney donor risk index to KDPI on the basis of the “Archived KDPI Mapping Tables,”^13^ both provided by the OPTN transplant HRSA website. Because KDPI was not introduced until more recently (2015) and mapping tables are limited to transplants occurring after 2010, this limited the range of historical data available. We performed all analyses using SAS version 9.4 (SAS, Cary, NC) and STATA version 17.0 (StataCorp, College Station, TX).

### Statistical Analysis

We applied a flexible parametric relative survival model^14^ with a time-varying covariate for waitlist year to extrapolate predictions beyond the observation period. In companion analyses, we compared predictions using this approach with other parametric and semiparametric models and found that the flexible parametric relative survival model was the most accurate. Flexible models resemble a Weibull model where the baseline hazard is flexibility fitted with cubic splines. By using a relative survival approach to our model, we provide some stability to the baseline hazard through incorporating data from the general population.^15^ This feature of the model is particularly useful in obtaining contemporary estimates where we need to extrapolate results beyond the observation period. To obtain relative survival estimates, we matched US life tables^16^ on age, sex, designated race, and year to our cohort and flexibly fitted the model with cubic splines as the baseline hazard. Thus, we estimated LYFT as the difference in life lost between transplant candidates and recipients:

$$\text{LYFT}=\text{Life}\;{\text{Lost}}_{\text{candidates}}-\text{Life}\;{\text{Lost}}_{\text{recipients}}$$

$$\text{Life}\;{\text{Lost}}_{\text{candidates}}={\text{mean}\;\text{expected}\;\text{survival}}_{\text{US}}-{\text{mean}\;\text{observed}\;\text{survival}}_{\text{candidates}}$$

$$\text{Life}\;{\text{Lost}}_{\text{recipients}}={\text{mean}\;\text{expected}\;\text{survival}}_{\text{US}}-{\text{mean}\;\text{observed}\;\text{survival}}_{\text{recipients}}$$

Note that the mean expected survival time in the general US population cancels from equation, leaving the difference in mean survival time between the patients receiving a transplant compared with dialysis. The mean expected survival time from the US population is mainly included to stabilize the predictions and adjust for demographic differences. We performed all analyses using SAS version 9.4 (Cary, NC) and STATA version 17.0 (College Station, TX).

This study was performed in accordance with the Declaration of Helsinki and was approved by the Stanford Institutional Review Board (protocol 40876). Owing to the nature of the research and absence of patient identifiers, the need for written informed consent was waived. The clinical and research activities being reported are consistent with the Principles of the Declaration of Istanbul as outlined in the Declaration of Istanbul on Organ Trafficking and Transplant Tourism. The data reported here have been supplied by the Hennepin Healthcare Research Institute as the contractor for the SRTR. The interpretation and reporting of these data are the responsibility of the author(s) and in no way should be seen as an official policy of or interpretation by the SRTR or the US Government.

## Results

**Figure 1:**
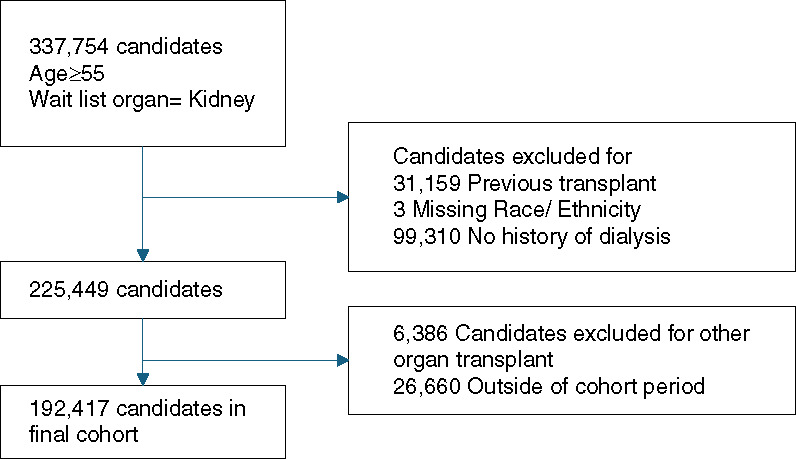
Cohort selection flow diagram.

Starting with the complete SRTR cohort (1987–2020), we selected the 337,754 candidates aged 55 and older listed for a kidney transplant. After exclusions described above, the analytic cohort consisted of 192,417 candidates (Figure 1), and 96,166 (50%) of these candidates received a kidney transplant. Fewer than 1% of the transplant candidates included in the analytic sample were listed before 1994.

We describe the differences at the time of listing among patients who received a transplant and those who remained on dialysis in Table 1. The results encompass the full cohort period of 1987–2020. The mean age and the proportion of patients above specific age thresholds increased over time. Most of the patients had diabetes as their primary diagnosis at the time of listing. The distribution of comorbidities was similar between groups. Twenty two percent of transplant recipients received a kidney from an ECD.

**Table 1:** Patient characteristics by treatment group (candidates on dialysis versus those that received a transplant)

Characteristic at the Time of Listing	All Candidates (*N*=192,417)	Candidates on Dialysis Only (*N*=96,251)	Transplant Recipients (*N*=96,166)
**Age group, yr**			
55–60	79,333 (41%)	36,547 (38%)	42,786 (45%)
61–65	56,868 (30%)	28,523 (30%)	28,345 (29%)
66–70	38,442 (20%)	21,016 (22%)	17,426 (18%)
Older than 71	17,774 (9%)	10,165 (11%)	7609 (8%)
Female	71,964 (37%)	36,597 (38%)	35,367 (37%)
**Race**			
Asian	13,614 (7%)	7325 (8%)	6289 (7%)
Hispanic^a^	32,100 (17%)	17,987 (19%)	14,113 (15%)
Non-Hispanic Black	55,050 (29%)	28,999 (31%)	26,051 (27%)
Non-Hispanic White	87,471 (45%)	38,593 (41%)	47,878 (50%)
Other^b^	4182 (2%)	2347 (2%)	1835 (2%)
**Listing year**			
1987–1995	5670 (3%)	2262 (2%)	3408 (4%)
1996–2000	21,854 (12%)	9776 (10%)	12,078 (13%)
2001–2005	30,618 (16%)	13,974 (14%)	16,644 (17%)
2006–2010	43,146 (23%)	22,414 (23%)	20,732 (22%)
2011–2015	48,828 (25%)	26,111 (27%)	22,717 (24%)
2016–2020	42,301 (22%)	21,714 (23%)	20,587 (21%)
**Primary diagnosis**			
Diabetes	91,225 (47%)	51,895 (54%)	39,330 (41%)
GN	19,812 (10%)	7405 (8%)	12,407 (13%)
Hypertension	46,576 (24%)	22,483 (24%)	24,093 (25%)
Other	34,696 (18%)	14,398 (15%)	20,298 (21%)
Missing (%)	108 (<1%)	70 (<1%)	38 (<1%)
**BMI**			
Mean (SD)	28.57 (5.25)	28.81 (5.37)	28.33 (5.11)
Missing (%)	3068 (2%)	1595 (2%)	1473 (2%)
**Candidate diagnosis**			
PVD			
*Yes*	19,660 (10%)	10,012 (10%)	9648 (10%)
*No*	166,775 (87%)	82,906 (86%)	83,869 (87%)
*Unknown*	5982 (3%)	3333 (3%)	2649 (3%)
Malignancy			
*Yes*	16,525 (9%)	7987 (8%)	8538 (9%)
*No*	171,245 (89%)	85,910 (89%)	85,335 (89%)
*Unknown*	4647 (2%)	2354 (2%)	2293 (2%)
COPD			
*Yes*	2927 (2%)	1675 (2%)	1252 (1%)
*No*	135,482 (70%)	66,932 (70%)	68,550 (71%)
*Unknown*	54,008 (28%)	27,644 (29%)	26,364 (27%)
CVD			
*Yes*	6134 (3%)	3491 (4%)	2643 (3%)
*No*	116,846 (61%)	56,030 (58%)	60,816 (63%)
*Unknown*	69,437 (36%)	36,730 (38%)	32,707 34%)
Diabetes			
*Yes*	110,143 (57%)	62,010 (64%)	48,133 (50%)
*No*	81,104 (42%)	33,567 (35%)	47,537 (49%)
*Unknown*	1170 (<1%)	674 (<1%)	496 (<1%)
**PRA**			
Mean % (SD)	4.60 (16.80)	4.89 (17.55)	4.31 (16.00)
Missing (%)	1040 (<1%)	151 (<1%)	889 (1%)
**Albumin, mg/dl**			
Mean (SD)	3.87 (0.56)	3.84 (0.56)	3.90 (0.56)
Missing (%)	41,420 (22%)	20,222 (21%)	21,198 (22%)
**Year of dialysis start**			
1987–1995	1328 (<1%)	740 (1%)	588 (1%)
1996–2000	13,233 (7%)	6513 (7%)	6720 (7%)
2001–2005	34,740 (18%)	16,339 (17%)	18,401 (19%)
2006–2010	43,208 (22%)	22,094 (23%)	21,114 (22%)
2011–2015	46,304 (24%)	24,097 (25%)	22,207 (23%)
2016–2020	28,770 (15%)	15,584 (16%)	13,186 (14%)
Missing	24,834 (13%)	10,884 (11%)	13,950 (15%)
**Dialysis modality**			
Hemodialysis	102,366 (53%)	505,524 (53%)	51,814 (54%)
Peritoneal dialysis	18,255 (9%)	8274 (9%)	9981 (10%)
Unknown	71,796 (37%)	37,425 (38%)	343,710 (36%)
**Characteristic at the time of transplant**			
ECD category		NA	
*Meets criteria*			20,931 (22%)
*Does not meet criteria*			56,670 (59%)
*Unknown*			18,565 (19%)
Donor type		NA	
*Deceased*			67,653 (70%)
*Living*			17,011 (18%)
*Missing*			11,502 (12%)
KDPI			
*<20%*		NA	3644 (2%)
*20%–85%*			30,645 (16%)
*>85%*			6154 (14%)
Missing^c^			151,974 (78%)

BMI, body mass index; COPD, chronic obstructive pulmonary disease; CVD, cerebral vascular disease; ECD, expanded criteria donor; KDPI, kidney donor profile index; NA, not applicable; PRA, panel-reactive antibody; PVD, peripheral vascular disease.

a Hispanic Black is <1% of the Hispanic race group.

b Other race includes Native American, Pacific Islander, and Multi-race.

c Tables for mapping scaled kidney donor risk index to KDPI were only available for 2010–2020.

**Figure 2:**
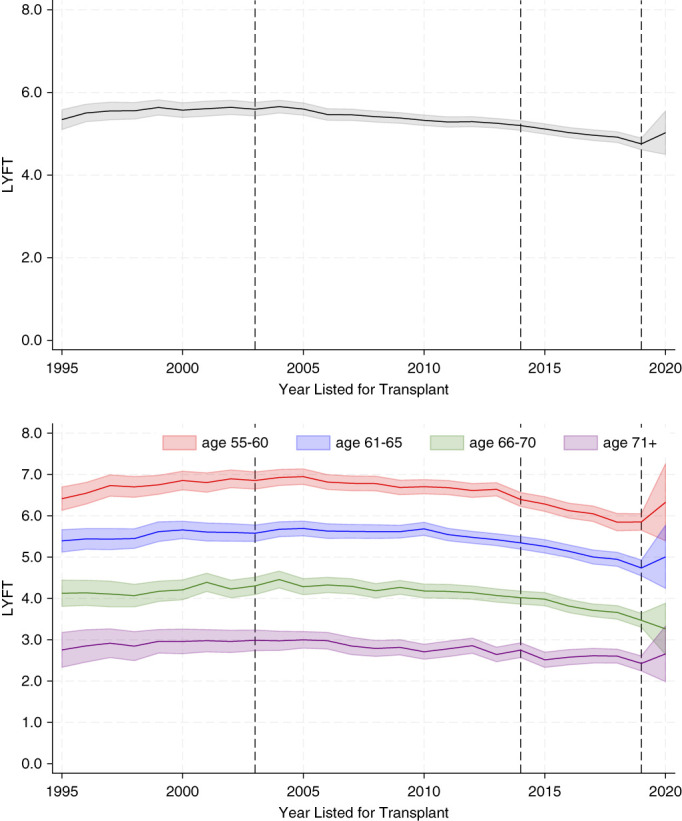
**Entire cohort's LYFT over time (top) and by age group (bottom).** Vertical lines indicate where major policy changes occurred. Shaded area is the 95% CI around the estimates. CI, confidence interval; LYFT, life years from transplant.

Figure 2 shows the crude average LYFT over time for the cohort from 1995 to 2020. LYFT declined slightly from 5.3 (95% CI, 5.1 to 5.6) years in 1995 to 4.8 (95% CI, 4.6 to 4.9) years in 2019 (top figure). The analysis stratified by age showed similar trends in each of the age groups, with younger ages having a slightly higher LYFT. The spike in LYFT after 2020 is an artifact of limited follow-up to observe the survival curve. Supplemental Table 1 provides the incidence of death censored graft loss for context. Our analyses included patients who received a living donor kidney. Supplemental Figure 1 shows the Figure 2 results stratified by living and deceased donor recipients.

**Figure 3:**
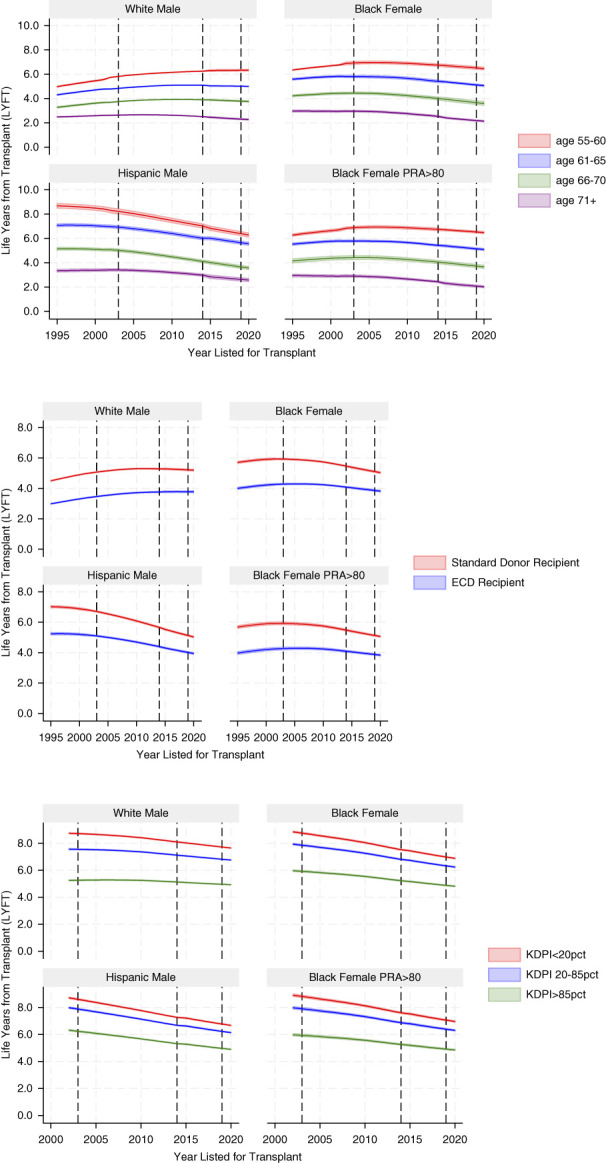
**Trends in LYFT over time by case and subgroup (age group and donor type).** Policy changes are denoted by vertical lines. Shaded area is the 95% CI around the estimates. ECD, expanded criteria donor; KDPI, kidney donor profile index; PRA, panel-reactive antibody.

In Figure 3, we simulated the projected LYFT for four example cases (quadrants 1–4) by year and age stratum (top), ECD (middle), and KDPI (bottom) using our model. For example, we project the LYFT for a White male with a BMI of 30–35, 1 year of dialysis, diabetes, a PRA of 0%–1%, and albumin of 3.5–4 mg from the model showing the trends by age group and year in the first quadrant (top). Younger age groups have higher LYFT than older age groups, standard donor kidney recipients have higher LYFT than ECD kidney recipients, and low KDPI kidney recipients have higher LYFT than high KDPI kidney recipients.

Policy shifts in 2002 (ECD) and 2015 (KDPI) are indicated by vertical lines. Trends over time are relatively flat. We note that the magnitude of gain in LYFT becomes smaller over time in three of the four cases, particularly for the Hispanic male. Nevertheless, we determined a positive difference of 2.0–6.5 years for all patients listed in 2020. Overall, we find a positive gain in LYFT for all cases and years examined. The survival advantage attributed to receiving a transplant over remaining on dialysis persists, regardless of age and donor status of the kidney received.

## Discussion

In this study of older kidney transplant candidates, we provide more contemporary estimates of life years gained using updated statistical methods. Our analytic approach yielded two major findings. First, we found that all groups of older kidney transplant candidates experience a gain in life years from kidney transplantation. Second, we found that in more recent years, the magnitude of this gain in LYFT has diminished, counter to what we had hypothesized. We suspect this trend is driven by changes in kidney transplant candidate characteristics and policies that have encouraged access to transplantation. In general, older adults who undergo kidney transplantation experience an increased lifespan relative to those who remain on dialysis—a finding that should be considered in future policy decisions and shared decision-making with kidney transplant candidates and their families. As we move into an era during which referral for kidney transplantation will be more strongly encouraged and kidney transplant candidates will likely be older in age, contemporary and highly accurate data should inform these critical decisions.

Other methods exist for estimating LYFT. Recently, Strohmaier *et al.* estimated the benefit of kidney transplantation over dialysis from the restricted mean survival time in a target trial of Austrian transplant candidates.^17^ They estimated the adjusted difference in 5-year and 10-year restricted mean survival time to be 0.5 and 2.4 years, respectively, in a predominately White population with an average age of 52 years. By contrast, we predicted an average of 6.3 years difference in lifetime for a non-Hispanic White man aged 55–60 years. The difference in our respective estimates is likely driven by country-specific transplantation policy, and the heterogeneity of the US kidney transplant candidate population, as well as our choice to use a flexible parametric relative survival model without a preset limit to follow-up duration. By contrast, Strohmaier *et al.* used Cox regression, which our group found overestimates predictions of mean survival time when compared with observed survival.^17^ While unified network for organ sharing death dates are reliable,^18^ the salmon effect^19^ (patients returning to their country of origin to die) may increase loss to follow-up in selected populations, such as those found in the United States, and result in over-estimates of survival for patients born outside of the United States.

Previously published studies parsed age as older and younger than 65 years, thereby sacrificing relevant information on differences in LYFT across a broader spectrum of older patients, an issue more relevant in 2024 than in 1999, when a much smaller proportion of kidney transplant candidates (and recipients) were within late-middle or older age groups.^1,20^ In this study, we provide a more refined picture of the benefit of transplantation. For persons 71 years or older, we show an average gain of 2 years from transplant. Notably, studies have shown transplanted candidates experience improved quality of life.^21^ The utility in transplanting patients older than 70 years, particularly given the scarcity of deceased donor organs, must be weighed against the benefit to older candidates in sufficiently good health.

Our updated LYFT estimates should be considered in the context of health policy changes to encourage use of marginal donor kidneys that expand the donor pool. Starting in 2003, such organs were designated as ECDs,^9^ a designation which was updated to high KDPI when the KDPI and the Kidney Allocation Score designations were added.^22^ Recipients who consent for these kidneys for the benefit of a possible shorter waiting time should be prepared for higher rates of delayed graft function and potentially inferior long-term allograft function compared with a standard KDPI offer. As testament to who is willing to accept these high KDPI offers, we note that 57% of waitlisted candidates aged 55 and over have consented (Tonya Eberhard, SRTR, personal communication, September 14, 2023). We suspect the more frequent use of less than optimal kidneys over time by older adults contributes to our finding that the gain in LYFT has attenuated somewhat in recent years. Another cause of the lower LYFT in more recent years is the decreased mortality for patients receiving dialysis.^23^ Nonetheless, our results show that transplantation remains unequivocally beneficial when compared with ongoing dialysis.

More recently, the Advancing American Kidney Health Executive Order signed in 2019 directed resources to encouraging kidney transplantation.^24^ This executive order financially incentivizes dialysis providers to refer and lead a growing share of patients to and through transplantation. The confluence of policies that promote transplantation will continue to increase the demand for kidney transplants among older adults.

Today's patients are older and more medically complex than 20 years ago.^25,26^ Wait times are longer, and more patients are willing to accept marginal organs. Given these changes in the candidate pool and available organs, one would expect a more dramatic decline in LYFT at the population level. However, we found that overall trends in LYFT remains relatively stable. We now have more granular data on marginal organs and transplantation technology has improved, enabling us to achieve better survival outcomes under suboptimal conditions. It is encouraging to find that there is a survival benefit for patients who receive marginal kidneys. Unfortunately, having more patients on the waitlist prolongs wait times and worse outcomes for patients that remain on dialysis. It is important that LYFT estimation methods keep pace with the current data to form more realistic expectations for the patient and the system.

Several limitations of our analysis should be noted. Generalizability of this study is limited to the population of patients with kidney failure on the waitlist with a history of requiring dialysis and does not include those who receive a preemptive transplant. For such individuals, we anticipate that the LYFT gained would be even greater. Our data also include kidney transplant candidates on the transplant list in 2019. Because we cannot fully observe the lifespan of such individuals, our trends are presumed from historical data. We also note that our results are derived from data using the historical eGFR formula including Black versus non-Black race, which is no longer endorsed. Because the use of the refit eGFR equation could result in earlier access to the waitlist by Black patients, the relative benefits in LYFT by race/ethnicity could change slightly. Our models are based on observational registry data, which are subject to unmeasured confounding and misclassification. While we included measured confounders available in the database, there is still the potential for selection and information bias that could contribute to inaccuracies in our LYFT estimate.

Prospective kidney transplant recipients and kidney transplant care providers need updated models using a contemporary cohort to guide current clinical practice, especially for older and more frail individuals who are more likely to accept and ultimately receive marginal organs. We anticipate the LYFT statistic will continue to be a useful metric to assess and communicate the benefits of transplant. Our update of the LYFT metric better represents the contemporary kidney transplant candidate population and will likely help stakeholders, including policymakers and payors, because they continue to assess the benefits of transplantation over dialysis, particularly in the use of marginal organs. However, we should be cautious when using survival measures to not allow these measures to bias selection of transplant candidates. We must strive for nuanced decision-making that accounts for changes in patient characteristics, health policy, and clinical advances over time.

## Supplementary Material

## Data Availability

Previously published data were used for this study. The rights to released data are retained by health and human services/HRSA and the SRTR Contractor. Information on how to access data is available upon reasonable request to the corresponding author.

## Supplemental Material

Supplemental Table 1. The percentage of observed graft failure of transplant patients in our cohort, by listing year.

Supplemental Figure 1. LYFT over time stratified by living and deceased donor recipients. Vertical lines indicate where major policy changes occurred. Shaded area is the 95% CI around the estimates.
