# Supplementary material for: Estimating the Benefit of Transplant Over Dialysis in Candidates Over 55 Years
Source: Kidney360. 2025 Jan 22;6(7):1198–206. doi: 10.34067/KID.0000000710 (PMC12338356; doi:10.34067/KID.0000000710)
Supplement: Supplementary file 1 [file kidney360-6-1198-s001.pdf]

ASN
American Society of Nephrology
JASN
Journal of the American Society of Nephrology
KIDNEY360
Accessing Our World From Every Angle
CJASN
Clinical Journal of the American Society of Nephrology

ASN Journal Disclosure Form

As per ASN journal policy, I have disclosed any financial relationships or commitments I have held in the past 36 months as included below. I have listed my Current Employer below to indicate there is a relationship requiring disclosure. If no relationship exists, my Current Employer is not listed.

P. Ahearn reports the following:
Employer: My spouse is employed by Google; and Ownership Interest: My spouse owns Google stock.

I understand that the information above will be published within the journal article, if accepted, and that failure to comply and/or to accurately and completely report the potential financial conflicts of interest could lead to the following: 1) Prior to publication, article rejection, or 2) Post-publication, sanctions ranging from, but not limited to, issuing a correction, reporting the inaccurate information to the authors' institution, banning authors from submitting work to ASN journals for varying lengths of time, and/or retraction of the published work.

Name: Patrick Ahearn
Manuscript ID: K360-2024-000318R2
Manuscript Title: Estimating the Benefit of Transplant Over Dialysis in Candidates Over 55 Years
Date of Completion: December 8, 2024
Disclosure Updated Date: December 8, 2024

ASN American Society of Nephrology | JASN Journal of the American Society of Nephrology | KIDNEY360 Accessing Our World From Every Angle | CJASN Clinical Journal of the American Society of Nephrology

ASN Journal Disclosure Form

As per ASN journal policy, I have disclosed any financial relationships or commitments I have held in the past 36 months as included below. I have listed my Current Employer below to indicate there is a relationship requiring disclosure. If no relationship exists, my Current Employer is not listed.

G. Chertow reports the following:
Employer: Stanford University School of Medicine; Consultancy: Akebia, Ardelyx, AstraZeneca, Beren, CalciMedica, Calico, Miromatrix, Panoramic, Sanifit, Toku, Unicycive, Vertex; Ownership Interest: Ardelyx, CloudCath, Durect, DxNow, Eliaz Therapeutics, Outset, Renibus, Unicycive; Research Funding: NIDDK, NIAID, CSL Behring; Advisory or Leadership Role: Board of Directors, Satellite Healthcare, Co-Editor, Brenner & Rector's The Kidney (Elsevier); and Other Interests or Relationships: DSMB service: NIDDK, Aethlon, Bayer, Mineralys, ReCor.

I understand that the information above will be published within the journal article, if accepted, and that failure to comply and/or to accurately and completely report the potential financial conflicts of interest could lead to the following: 1) Prior to publication, article rejection, or 2) Post-publication, sanctions ranging from, but not limited to, issuing a correction, reporting the inaccurate information to the authors' institution, banning authors from submitting work to ASN journals for varying lengths of time, and/or retraction of the published work.

Name: Glenn M. Chertow
Manuscript ID: K360-2024-000318R2
Manuscript Title: Estimating the Benefit of Transplant Over Dialysis in Candidates Over 55 Years
Date of Completion: November 30, 2024
Disclosure Updated Date: June 18, 2024

ASN American Society of Nephrology JASN Journal of the American Society of Nephrology KIDNEY360 Accessing Our World From Every Angle CJASN Clinical Journal of the American Society of Nephrology

ASN Journal Disclosure Form

As per ASN journal policy, I have disclosed any financial relationships or commitments I have held in the past 36 months as included below. I have listed my Current Employer below to indicate there is a relationship requiring disclosure. If no relationship exists, my Current Employer is not listed.

C. Liu reports the following:
Employer: Stanford University School of Medicine

I understand that the information above will be published within the journal article, if accepted, and that failure to comply and/or to accurately and completely report the potential financial conflicts of interest could lead to the following: 1) Prior to publication, article rejection, or 2) Post-publication, sanctions ranging from, but not limited to, issuing a correction, reporting the inaccurate information to the authors' institution, banning authors from submitting work to ASN journals for varying lengths of time, and/or retraction of the published work.

Name: Christine Liu
Manuscript ID: K360-2024-000318R1
Manuscript Title: Estimating the Benefit of Transplant Over Dialysis in Candidates Over 55 Years
Date of Completion: August 25, 2024
Disclosure Updated Date: August 23, 2024

ASN American Society of Nephrology
JASN Journal of the American Society of Nephrology
KIDNEY360 Accessing Our World From Every Angle
CJASN Clinical Journal of the American Society of Nephrology

ASN Journal Disclosure Form

As per ASN journal policy, I have disclosed any financial relationships or commitments I have held in the past 36 months as included below. I have listed my Current Employer below to indicate there is a relationship requiring disclosure. If no relationship exists, my Current Employer is not listed.

M. Stedman has nothing to disclose.

I understand that the information above will be published within the journal article, if accepted, and that failure to comply and/or to accurately and completely report the potential financial conflicts of interest could lead to the following: 1) Prior to publication, article rejection, or 2) Post-publication, sanctions ranging from, but not limited to, issuing a correction, reporting the inaccurate information to the authors' institution, banning authors from submitting work to ASN journals for varying lengths of time, and/or retraction of the published work.

Name: Margaret R. Stedman
Manuscript ID: K360-2024-000318R2
Manuscript Title: Estimating the Benefit of Transplant Over Dialysis in Candidates Over 55 Years
Date of Completion: December 2, 2024
Disclosure Updated Date: December 2, 2024

ASN American Society of Nephrology | JASN Journal of the American Society of Nephrology | KIDNEY360 Accessing Our World From Every Angle | CJASN Clinical Journal of the American Society of Nephrology

ASN Journal Disclosure Form

As per ASN journal policy, I have disclosed any financial relationships or commitments I have held in the past 36 months as included below. I have listed my Current Employer below to indicate there is a relationship requiring disclosure. If no relationship exists, my Current Employer is not listed.

J. Tan reports the following:
Employer: Stanford University - Self; GNE/Roche - Spouse; Ownership Interest: GNE/Roche - Spouse; and Advisory or Leadership Role: Am J Transplantation AE; UpToDate Kidney Transplant Section Editor.

I understand that the information above will be published within the journal article, if accepted, and that failure to comply and/or to accurately and completely report the potential financial conflicts of interest could lead to the following: 1) Prior to publication, article rejection, or 2) Post-publication, sanctions ranging from, but not limited to, issuing a correction, reporting the inaccurate information to the authors' institution, banning authors from submitting work to ASN journals for varying lengths of time, and/or retraction of the published work.

Name: Jane C. Tan
Manuscript ID: K360-2024-000318R2
Manuscript Title: Estimating the Benefit of Transplant Over Dialysis in Candidates Over 55 Years,"
Date of Completion: January 10, 2025
Disclosure Updated Date: January 10, 2025
