# Supplementary material for: Estimating the Benefit of Transplant Over Dialysis in Candidates Over 55 Years
Source: Kidney360. 2025 Jan 22;6(7):1198–206. doi: 10.34067/KID.0000000710 (PMC12338356; doi:10.34067/KID.0000000710)

SUPPLEMENT

Supplemental Table 1: The percentage of observed graft failure of transplant patients in our cohort, by listing year.

Listing Year	N	% Graft failure	% Deaths	Median follow-up time (years) from listing date
1987-1995	3407	27.2%	93.3%	9.5
1996-2000	12078	26.2%	88%	10.1
2001-2005	16638	23.7%	72.9%	11.3
2006-2010	20728	18.3%	47.4%	10.0
2011-2015	22715	10.2%	16.3%	6.2
2016-2020	20587	5.2%	3.1%	3.8

Supplemental Figure 1: LYFT over time stratified by living and deceased donor recipients. Vertical lines indicate where major policy changes occurred. Shaded area is the 95% confidence interval around the estimates.

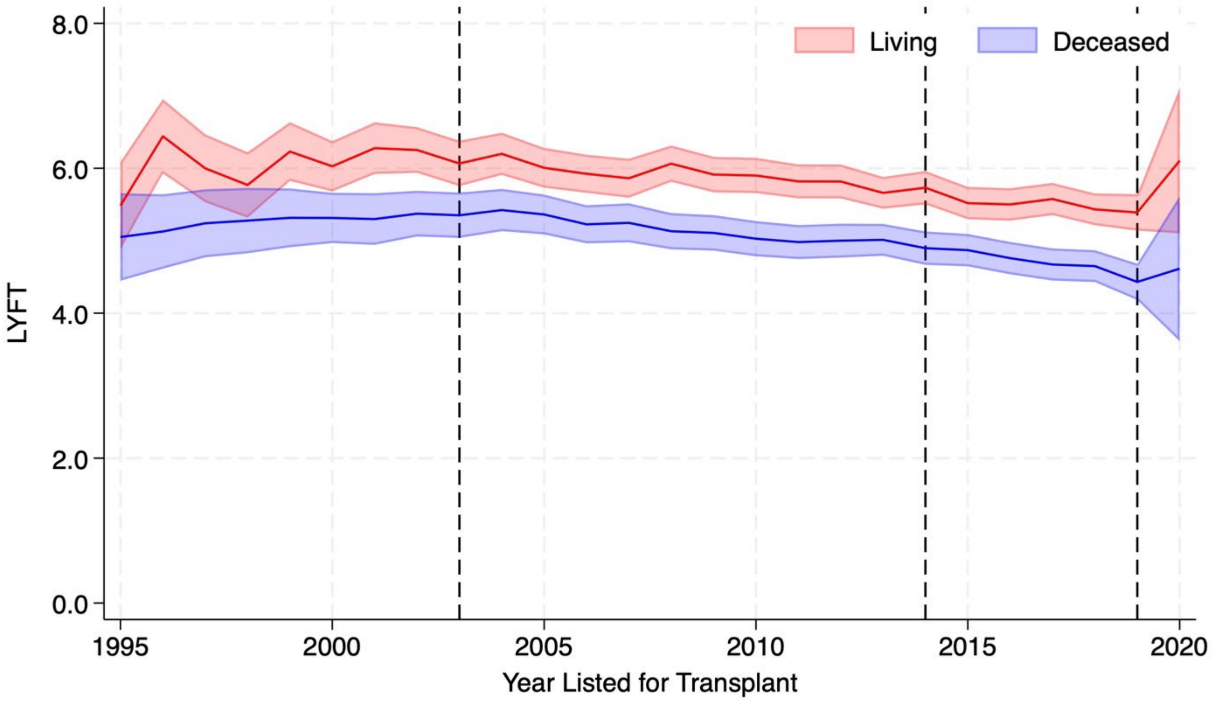

Supplement: Supplementary file 2 [file kidney360-6-1198-s002.pdf]
